# A Cognitive Apprenticeship-Based Faculty Development Intervention for Emergency Medicine Educators

**DOI:** 10.5811/westjem.2017.11.36429

**Published:** 2017-12-18

**Authors:** Chris Merritt, Michelle Daniel, Brendan W. Munzer, Mariann Nocera, Joshua C. Ross, Sally A. Santen

**Affiliations:** * Alpert Medical School of Brown University, Departments of Emergency Medicine and Pediatrics, Providence, Rhode Island; † University of Michigan Medical School, Department of Emergency Medicine, Ann Arbor, Michigan; ‡ University of Connecticut School of Medicine, Departments of Pediatrics and Emergency Medicine, Division of Pediatric Emergency Medicine, Farmington, Connecticut; § University of Wisconsin School of Medicine and Public Health, Department of Emergency Medicine, Madison, Wisconsin; # Virginia Commonwealth University School of Medicine, Richmond, Virginia

## Abstract

In just a few years of preparation, emergency medicine (EM) trainees must achieve expertise across the broad spectrum of skills critical to the practice of the specialty. Though education occurs in many contexts, much learning occurs on the job, caring for patients under the guidance of clinical educators. The cognitive apprenticeship framework, originally described in primary and secondary education, has been applied to workplace-based medical training. The framework includes a variety of teaching methods: scaffolding, modeling, articulation, reflection, and exploration, applied in a safe learning environment. Without understanding these methods within a theoretical framework, faculty may not apply the methods optimally. Here we describe a faculty development intervention during which participants articulate, share, and practice their own applications of cognitive-apprenticeship methods to learners in EM. We summarize themes identified by workshop participants, and provide suggestions for tailoring the application of these methods to varying levels of EM learners. The cognitive-apprenticeship framework allows for a common understanding of the methods used in clinical teaching toward independence. Clinical educators should be encouraged to reflect critically on their methods, while being offered the opportunity to share and learn from others.

## BACKGROUND

Emergency medicine (EM) trainees must achieve expertise across the broad spectrum of clinical skills critical to EM practice, achieving competence in only a few short years. While EM training includes didactics, self-directed learning, and periodic assessments, the key learning occurs while caring for patients under the supervision of experienced physicians. While early medical education often focuses on transmission and retention of data, learners must ultimately gain practical experience applying clinical reasoning, learning to work in teams, and approaching complicated problems and procedures. The understanding and strategic implementation of problem-solving strategies, heuristic approaches, and metacognitive skills leads to the type of understanding that allows the novice to become the expert.

The passing on of both domain and strategic knowledge happens through a process that other professions call an *apprenticeship*: a skill is first observed, then taught, and then practiced until mastery is achieved. To teach the art of medicine, however, we must also model and develop a set of cognitive skills. To do this requires a *cognitive apprenticeship*.

Introduced in the elementary and secondary education literature in the 1980s,^1^,^2^ the cognitive-apprenticeship framework has been introduced into the medical education lexicon by Stalmeijer and others.^3^ In a cognitive apprenticeship, the expert provides access to cognitive strategies and skills and opportunities to explore. Cognitive apprenticeship encompasses the content, the sequential ordering of learning activities (increasingly diverse, increasingly complex), and the social characteristics of the community of practice.^4^

The overarching process of a cognitive apprenticeship is to provide *scaffolding* – just enough structural support that novices may begin to build their own skills and strategies. As a novice’s skills solidify, this scaffolding is removed, until eventually trainees are able to practice independently. The cognitive apprenticeship includes a number of methods for providing cognitive supports, many of which may be familiar to teachers and learners in EM^5^:

- **Modeling**- Experts model the traits and behaviors they would like to see reflected in their learners. Experts make explicit what they intend to demonstrate; if they don’t, learners may make mistaken assumptions. Modeling is a constant process. Successful modeling builds the foundation for increasing cognitive independence.
- **Coaching**- A clinical educator prepares his or her team for what to expect, makes adjustments based on the circumstances, provides guidance and feedback in real time, serves as motivator, mentor, and at times a taskmaster.
- **Articulation**- A learner must be able to articulate clinical reasoning so that educators may be sure that the understanding is complete. Experts must also articulate their own understanding, which may prove difficult once the processes have become automatic.
- **Reflection**- Reflection may not be automatic to some; educators should encourage learners to consider what an encounter has taught them, how their future approach could change, and how to apply what they’ve experienced to new problems.
- **Exploration**- Learners must be given room for exploration. True learning happens just beyond the boundaries of what’s comfortable; learners should be encouraged to push those boundaries. Educators should urge learners to set goals to overcome weaknesses and build on strengths, and to regularly re-evaluate these goals in the context of new learning and new experience.

Though some educators may use these methods intuitively, most teachers in EM and other disciplines do not receive training in a theoretical understanding of clinical supervision. We believe that by understanding a theoretical framework and by being intentional in its application, educators may provide more effective scaffolding upon which learners may construct their working knowledge. With this goal in mind, this educational advance reviews the cognitive-apprenticeship model and its application to EM. Methods and materials are provided so that EM educators can run workshops with their faculty to train them in this method.

## OBJECTIVES

We describe an interactive faculty development workshop designed to provide both an introduction to the cognitive-apprenticeship framework and an opportunity to reflect on its application in EM training. Stated objectives were the following:

- Compare a variety of techniques for providing cognitive support in the context of apprenticeship teaching and learning
- Anticipate the cognitive supports required by learners as they seek and attain entrustment
- Have discussed several techniques of cognitive support that can be incorporated into educational practice.

## CURRICULAR DESIGN

We facilitated a series of faculty development workshops over the course of 2015–2017, including sessions aimed specifically at EM faculty at three consecutive annual meetings of the Society for Academic Emergency Medicine (SAEM), as well as sessions for multi-specialty teaching faculty at medical schools of the University of Michigan and Brown University. Each time the workshop was revised, primarily for ease of understanding and appropriateness of visual aids and visual content, based on feedback. The final version is described here. In one instance, participants received a brief publication in advance describing the cognitive-apprenticeship framework. This was not possible in other workshop settings.

**Figure 1:**
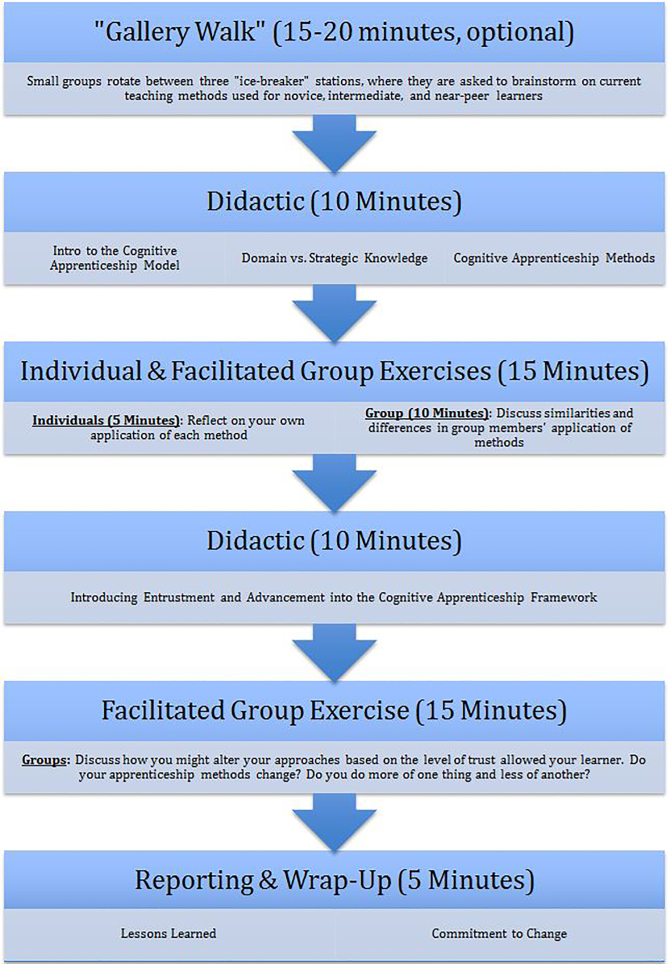
Schematic of the faculty development program, scaled for a one-hour presentation. Materials used for the didactic presentation, including a script and slides, are available from the authors upon request.

An initial “Gallery Walk” activity (if time allows) serves to set the stage, activating participants’ prior knowledge and identifying themes for facilitators to highlight throughout the discussion (Figure 1). At a series of stations, participants discussed teaching methods used in daily practice. Assuming that participants had no familiarity with the cognitive-apprenticeship model, a brief didactic presentation then laid out the scope of the model and provided working definitions for its methods so that participants could work within a shared framework using shared language.

Didactic elements were punctuated by individual reflection and small-group discussion among groups of 6–8 participants, each with 1–2 facilitators. In the first, participants were asked to reflect briefly on the application of these methods in their current educational practices, followed by facilitated group discussion comparing participants’ experiences.

A second brief didactic element describes one framework for understanding learner advancement based on levels of entrustment. Participants worked in groups to discuss how these methods might be adjusted for learners of varying levels of expertise, suggesting revisions to the cognitive-apprenticeship model as initially presented. Using an informal debriefing process following each workshop, group facilitators identified broad themes from the resultant discussions. These themes, collected over successive iterations of this educational advance, have been tabulated for presentation here (Table), and are now provided as “talking points” to assist faculty facilitators in this workshop.

## IMPACT AND EFFECTIVENESS

**Figure 2:**
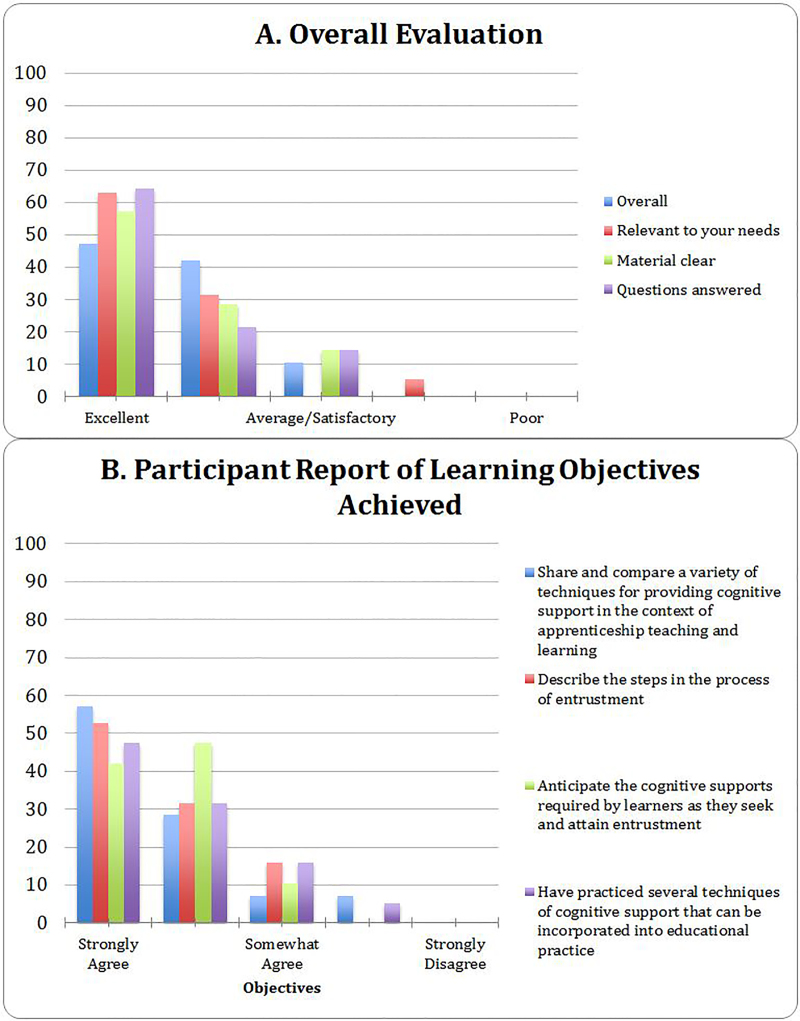
Participant evaluation of a cognitive apprenticeship-based faculty development workshop. Global evaluation of satisfaction (A) and achievement of learning objectives (B) during two early iterations of the faculty development workshop, reported as percentage of total respondents (n=19).

Nearly 90 participants have been estimated to have taken part in this series of faculty development sessions during 2016–2017. (Exact participation data was not available from the SAEM conferences.) Participants included resident learners as well as junior and senior faculty members. Overall evaluation from a subset of participants participating in the local, multi-specialty faculty development workshop presentations are shown in Figure 2.

Participants reported applying combinations of cognitive-apprenticeship methods depending upon the setting, timing and degree of experience of learners. We were able to identify broad trends in how the methods were applied by participants based on the level of learner being taught (Table). In narrative feedback, participants reported that by making the theoretical framework explicit, they recognized opportunities to be more intentional with their choices of methods and timing, and were better prepared to adjust their methods to better suit the needs of their learners.

## DISCUSSION

As learners negotiate the path from novice to expert, the clinician educator’s role is to guide that path. As the teaching of the clinical practice of medicine remains very much an apprenticeship, the application of the cognitive-apprenticeship model has been shown to be acceptable to learners and educators alike, and indeed has been widely applied even if not explicitly named or recognized.^3^,^6^–^8^

We have described here a faculty development intervention aimed at helping educators reflect on and make explicit their understanding and application of the cognitive-apprenticeship model in EM education. From the workshop discussions, we have identified a number of patterns in how these methods are applied to education practice, varying depending on the level of learner involved:

- Novice learners will require the greatest degree of scaffolding. Novices learn from observation, coaching, and early articulation. Teachers model a range of approaches. Beyond demonstration, the expert must also articulate *why* he or she is focusing on certain elements. The novice learner can be coached to develop schema for various illness scripts, beginning to identify nuanced patterns. Reflection and exploration may be limited, but are important to gauging learner reactions, planning, and goal-setting. As learners develop, they will be able to apply these lessons to new and unfamiliar situations.
- For mid-level learners, workshop participants suggest that the focus of modeling shift from managing individual performance to managing the medical team. Setting a positive and collaborative “tone” between members of the patient care team is an important lesson. Likewise, the coach may begin to focus on action plans. As mid-level learners explore management approaches, real-time feedback allows the learner to achieve increasing competency. The coach also has a responsibility to motivate and challenge mid-level learners, providing learning opportunities within their zone of proximal development, encouraging reflection on how past experiences may influence present and future experiences.
- Near-independent learners can benefit from modeling of effective strategies to manage the healthcare system and advocate for patients. Effectively handling difficult conversations as well as directing care for patients with complex needs is the hallmark of the expert. Explicitly modeling how to listen and understand a patient’s needs, advocate appropriately and “close the loop” with multiple partners will advance trainees’ professional growth. The coach serves as mentor, sponsor, and advocate for the learner. Reflecting upon problems and strategies in a safe, supportive environment promotes continued lifelong learning.

This application of cognitive-apprenticeship strategies is consistent with others’ observations. Examination of learners’ preferences shows that teaching practices should evolve along with learners’ degree of development, initially focusing more on the role of the supervisor, before gradually letting learners take charge.^9^ The presence of a safe learning environment proves crucial, and learners respond to increasing independence differently depending on their sense of support.^10^

Implementation of this faculty development intervention requires that facilitators become familiar with the cognitive apprenticeship framework. Review of a few key pieces of literature may be sufficient to prepare to implement this in a local faculty development program.^4^,^6^,^9^ The interactive nature of the discussion groups – which form the richest portion of the experience – may require only that facilitators are prepared with a few key questions to spur conversation or redirect discussions. Based on our experience, small groups positioned around a table work best with no more than eight discussants and at least one facilitator.

Groups have held rich discussions with both single-specialty or multi-specialty composition, though the direction of conversation may differ among these groups. In most cases, our presentations have been limited to one hour, though by allowing longer periods for group discussion it can easily be adapted to 90 minutes without substantial revision. In two presentations, an additional component – a “gallery walk” – was used as an opening ice-breaker (Figure 1), allowing participants to share practices and experience, and activating prior knowledge on which the group can build.

The true impact of the innovation presented here is significantly limited by the availability of reliable outcome data. Evaluation questions measured the participants’ subjective responses rather than behavior changes as a result of the workshop. Stronger evidence for the effectiveness of the workshop could be obtained through more rigorous longitudinal assessment of participants. The evaluations reported are from the early iterations only, and similar data from the most recent presentations is not available. However, each presentation has stimulated rich discussion among clinician educators, and the format has not been altered significantly.

## CONCLUSION

The methods and framework of the cognitive-apprenticeship model are recognized and accepted by clinical educators in emergency medicine. By providing an opportunity to articulate, reflect on, and explore the application of these methods in a safe learning environment of fellow educators in the faculty development setting, we have shown that rich discussions and sharing of strategies can be achieved. Clinical educators should be encouraged to reflect critically on their methods, while being offered the opportunity to share and learn from others. The cognitive-apprenticeship framework allows for a common understanding of the methods used in clinical teaching toward independence.

## Figures and Tables

**Table:** Application of cognitive-apprenticeship methods to varying levels of learners. From discussions held during several successive workshops, we identified several themes in educators’ application of the cognitive-apprenticeship teaching methods. While the definition of learner levels (novice, mid-level, near-independent) is highly dependent on the learning context (a “novice” may be a preclinical medical student in one context or a first-year fellow in training in another context), the application of techniques may be adapted to each context.

Cognitive apprenticeship method	Description of teacher – learner interaction	Novice learner	Mid-level learner	Near-independent learner
Modeling	Expert performs a task so that learner can observe; the expert explains heuristics and control processes used in applying basic conceptual and procedural knowledge.	Teaching/learning by observation Example: Perform an H&P on a patient Expert explains rationale behind specific actions	Set the tone, always Foster engagement in the healthcare team Expert explicitly models: team communication advanced patient care skills	Expert demonstrates and debriefs system-level skills: optimizing resources collaborating with consultants handling difficult patient interactions
Coaching	Expert prepares or observes learner during task performance and offers hints, scaffolding, feedback, reminders and new tasks aimed at bringing the learner’s performance closer to expert performance.	Help learners anticipate interactions using teaching scripts Emphasize important considerations	Challenge learners to improve Provide guided practice Give actionable feedback, hints, and reminders	Provide a safe learning environment for theoretical discussions Provide mentorship and advocacy to develop lifelong learning
Articulation	Both learner and teacher verbalize internal thought processes, focusing on the why in addition to the what.	Articulate domain knowledge, basic medical reasoning Teachers reinforce strengths, fill gaps Use probing questions to diagnose the learner	Articulate more advanced reasoning, providing support for actions Teachers recognize multiple approaches, verbalize advantages of one over another	Articulate systems-related processes or global thinking Anticipate future needs of patients and systems Plan prevention strategies
Reflection	Learners are encouraged to reflect on their own skills, for example in problem-solving or human interaction, as a means to identifying goals for improvement or change.	Reflect on learner’s own reactions (e.g. “How did that make you feel? Why do you think you had that emotional response?”)	Reflect on how interactions are influenced by previous experiences (e.g. “You’ve seen patients with this before. How can you improve on your management?”)	Reflect on managing increasingly complex problems, using “what if?” questions.
Exploration	Learners develop their own learning goals, and begin to develop strategies to achieve these goals.	Explore general concepts or learning goals for discrete problems or complaints	Explore different management styles, even if the “path” differs from what the expert has in mind	Explore management strategies with little supervision or support, mirroring true independence

*H&P,* history and physical.
